# Prediction of Surface Roughness as a Function of Temperature for SiO_2_ Thin-Film in PECVD Process

**DOI:** 10.3390/mi13020314

**Published:** 2022-02-17

**Authors:** Muhammad Rizwan Amirzada, Yousuf Khan, Muhammad Khurram Ehsan, Atiq Ur Rehman, Abdul Aleem Jamali, Abdul Rafay Khatri

**Affiliations:** 1 Faculty of Engineering and Computer Sciences, National University of Modern Languages, Islamabad 44000, Pakistan; 2 Department of Electronic Engineering, Balochistan University of Information Technology, Engineering and Management Sciences, Quetta 87300, Pakistan; yousuf.khan@buitms.edu.pk (Y.K.); atiqkhantareen@gmail.com (A.U.R.); 3 Faculty of Engineering Sciences, Bahria University, Lahore Campus, 47-C, Civic Center, Johar Town, Lahore 54000, Pakistan; mehsan.bulc@bahria.edu.pk; 4 Department of Electronic Engineering, Quaid-e-Awam University of Engineering, Science and Technology, Nawabshah 67480, Pakistan; jamali.abdulaleem@quest.edu.pk (A.A.J.); arkhatri@quest.edu.pk (A.R.K.)

**Keywords:** surface roughness, PECVD process, SiO_2_ thin-films, analytical prediction, MEMS, micro-mirrors

## Abstract

An analytical model to predict the surface roughness for the plasma-enhanced chemical vapor deposition (PECVD) process over a large range of temperature values is still nonexistent. By using an existing prediction model, the surface roughness can directly be calculated instead of repeating the experimental processes, which can largely save time and resources. This research work focuses on the investigation and analytical modeling of surface roughness of SiO_2_ deposition using the PECVD process for almost the whole range of operating temperatures, i.e., 80 to 450 °C. The proposed model is based on experimental data of surface roughness against different temperature conditions in the PECVD process measured using atomic force microscopy (AFM). The quality of these SiO_2_ layers was studied against an isolation layer in a microelectromechanical system (MEMS) for light steering applications. The analytical model employs different mathematical approaches such as linear and cubic regressions over the measured values to develop a prediction model for the whole operating temperature range of the PECVD process. The proposed prediction model is validated by calculating the percent match of the analytical model with experimental data for different temperature ranges, counting the correlations and error bars.

## 1. Introduction

**Figure 1:**
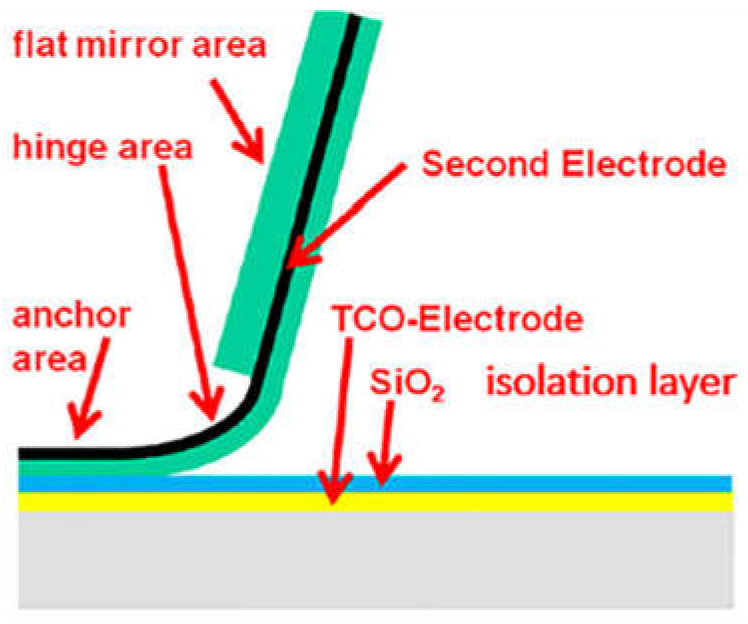
Schematic diagram of a micromirror implemented on a float glass substrate with bottom electrode shown in yellow layer, isolation layer in blue and aluminum-based actuating electrode in green color [6].

Microelectromechanical systems (MEMSs) are the combination of the electrical and mechanical systems at the micro level to form tiny integrated devices. Recently, MEMSs have been utilized in a number of sensing applications, i.e., pressure sensing [1], gas sensing [2], temperature sensing [3], chemical sensing [4] and magnetic sensing [5], due to their small size and high mechanical efficiency. Correspondingly, MEMSs are used for flow control in numerous systems such as micropumps, airfoils and optical switches [6]. MEMSs are also useful in micromirror arrays, implemented in window glasses for guiding the daylight to the desired area inside the buildings, as shown in Figure 1. 

**Figure 2:**
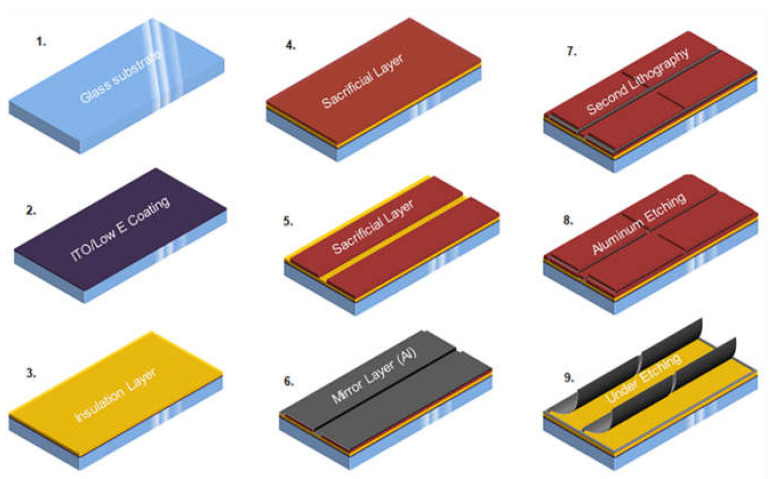
Steps involved in the fabrication process of the MEMS-based micromirrors [7].

The light can be steered by changing the angle of the metallic foils inside the array by electrical actuation. The fabrication steps during the process of the MEMS-based micromirrors are reflected in Figure 2.

**Figure 3:**
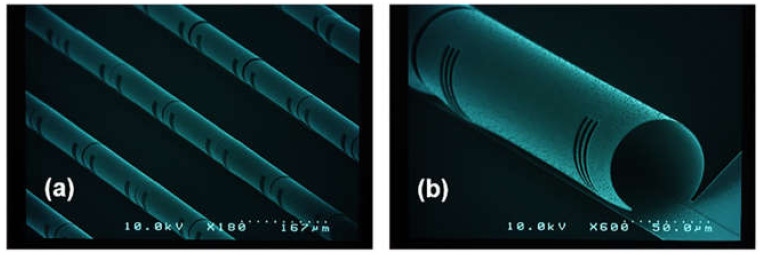
SEM photos of the fabricated structures of the MEMS-based micromirrors: (**a**) array; (**b**) single.

Aluminum (Al) is generally used in micromirrors as a reflectance layer due to its properties such as good electrical conductivity, high reflectivity, corrosion resistance [8,9] and low wear resistance [10,11,12]. To realize electrical actuation, the MEMS micromirror system consists of two electrodes separated by an isolation layer. The isolation layer mostly comprises silicon dioxide (${\mathrm{SiO}}_{2}$), which is the first choice due to its benefits such as providing good insulation between two conducting layers, easy availability, low cost, transparency and high mechanical flexibility [13,14,15,16,17]. The actual fabricated structure of the MEMS-based micromirrors is shown in Figure 3.

To ensure good insulation properties and high transparency, the quality of the deposited SiO_2_ layer becomes very crucial. Hence, during the deposition process of the ${\mathrm{SiO}}_{2}$ for MEMS-based devices, the surface roughness is precisely taken into account as it greatly affects the performance, reliability and isolation of the system [18]. Moreover, it heavily depends upon the deposition rate, temperature and thickness of the layers, as in the case of the solar cells, acting as the main component of photovoltaics [19,20,21,22]. The thin-film deposition techniques used for SiO_2_ deposition include sputter deposition method, thermal evaporation, chemical vapor deposition (CVD), ion beam deposition (IBD) [23], physical vapor deposition (PVD) and PECVD. Among the above-mentioned technologies, the PECVD process is the most widely used deposition technique since it offers a variety of advantages such as low operating temperatures and cost-effectiveness. PECVD processes offer a wide operating temperature range from 60 to 300 °C with control of the thickness and surface roughness of the layers [24,25,26]. Additionally, the PECVD process allows deposition of the industrial-scale high-quality layers with homogeneity and adherence [27]. It is known from the research [28,29,30] that the surface roughness of the deposited layers is greatly affected by the temperature inside the chamber during the process. A detailed investigation on variation in the deposition temperature of the PECVD process for the ${\mathrm{SiO}}_{2}$ layers to emulate the changes in the surface roughness is presented in [6].

Moreover, among analytical modeling techniques [31,32], machine learning is one of the leading fields in this modern era, finding application in a variety of areas such as automation of machines, robotics, nanophononics [33] and embedded systems [34]. In nanotechnology, machine learning algorithms can be used to predict the response of a particular process or structural parameters using the estimation from the available data as in the prediction of the surface roughness [35,36,37]. Henceforth, in this research, two machine learning algorithms, i.e., linear [38] and cubic regressions [39], are taken to predict the surface roughness against the change in the temperature during the PECVD deposition technique to provide an initial idea about the surface roughness without consuming the resources and time of researchers. By using this model, one can easily predict the value of surface roughness for the PECVD process at a certain temperature.

### 1.1. Linear Regression

Linear regression has been widely used in the field of machine learning to estimate the response of the system for the missing data from the available information. It comprises independent and non-independent variables and uses a combination of the input variables $\left(x\right)$ to predict the output variable $\left(y\right)$ and is imitated by Equation (1):

(1) y=a+b⋅x

where ‘a’ is the bias coefficient and ‘b’ is the coefficient of the input variable ‘x’ (slope).

### 1.2. Cubic Regression

Cubic regression is the third-degree equation used to predict the response of a system. It is also a widely used machine learning technique nowadays to predict the behavior of a system or trend in the data values to estimate the missing data. Equation (2) represents the general expression for cubic regression:

(2) $$y=ax^{3}+bx^{2}+cx+d$$

where a, b, and c are the coefficients and d is the *y*-intercept.

## 2. Experiment

In the first step of substrate preparation, the surface of the specimen was treated with isopropyl alcohol and pure nitrogen flow as rinsing and drying agents, respectively. In the second step, a 150 nm thick layer of ${\mathrm{SiO}}_{2}$ was deposited on the specimen, which is considered to be optimum for various devices. The deposition of the SiO_2_ isolation layer was carried out using the PECVD process in accordance with deposition parameters given in Table 1. The specimen was allowed into the heated chamber along with the silane gas and oxygen in presence of high energy plasma with values in standard cubic centimeters per minute (sccm) as mentioned in Table 1. The process is carried out in presence of nitrogen gas and a typical vacuum pressure of 1 torr. Typically, two temperature values, i.e., 120 and 300 °C, are used for the deposition procedures. However, the chamber temperature was varied from 80 to 300 °C in this research work to investigate the effects of temperature on surface roughness of the deposited layers using high frequency (HF) and low frequency (LF) sources.

After the deposition of the SiO_2_ layers, the surface roughness was investigated using atomic force microscopy (AFM), and Gwyddion software was used for the images. The AFM technology comprises three different surface profiling modes, i.e., contact mode, noncontact mode and tapping mode [40]. To avoid adhesion and shear forces problems arising in the contact mode [41,42], tapping mode was used for surface profiling. The average value of surface roughness [43] was used to determine the surface roughness of the layers against the given temperatures, defined by Equation (3) as

(3) $$R_{a}=\frac{1}{L}\int_{0}^{L}\left|\;Y\left(x\right)\right|dx$$

where $R_{a}$ represents surface roughness, Y is the total area and L is the total number of points used for the calculation of the surface roughness. 

## 3. Results and Discussion

The PECVD system allows the temperature of the substrate holder to be changed during the process, providing the opportunity to examine the behavior of the surface roughness against different applied temperatures. The experimental values for variation in the PECVD process temperature ranged from 80 to 300 °C in steps of 10, 20, 30 and 40 °C, and resulting surface roughness (nm) is shown in Table 2.

**Figure 4:**
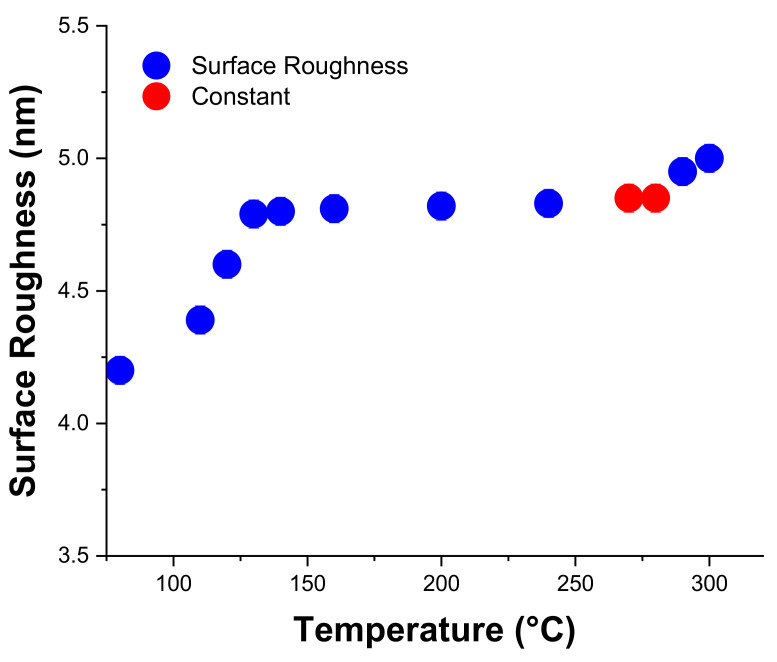
Graph representing surface roughness against the variation in the temperature using the PECVD process.

To graphically observe the variation in surface roughness as a function of PECVD process temperature, the experimental readings shown in Table 2 are plotted in Figure 4.

In Figure 4, it can be seen that the value of the surface roughness changes abruptly with the change in the temperature from 80 to 130 °C, representing maximum change. From 130 to 160 °C, the change in the value of the surface roughness is minimum, reflecting slow variation. Likewise, from 200 to 270 °C, the surface roughness increases consistently with the increase in the temperature. Consequently, from 270 to 280 °C, the value of the surface roughness remains constant. Onwards, with the increase in the temperature from 280 to 300 °C, the value of the surface roughness tends to increase congruently. 

During the PECVD process, the silane gas and oxygen are ionized in presence of the HF plasma inside the chamber to form ${\mathrm{SiO}}_{2}$ molecules and fall down to the substrate surface. These deposited molecules are loosely attached to the surface of the substrate and eventually fix to the surface in presence of high temperatures. Consequently, this movement of clusters increases the surface diffusion length l, which in turn increases surface roughness, and the process is known as surface migration. Moreover, the surface diffusion length is given by Equation (4):

(4) $$L=\sqrt{D}\tau$$

where D is the material-dependent diffusion constant having values in direct proportion to the values of the temperature and τ is equivalent to the deposition time of one layer.

Similarly, an added reason for the increase in the value of the surface roughness is the initial cluster size, formed at the start of the deposition process. Apart from this, the other reason for the increase in the surface roughness is the collision of two clusters having motion proportional to the substrate temperature forming one larger cluster.

To predict the surface roughness values for operating temperature beyond experimental values, i.e., 300 °C, the data were processed using different machine learning approaches based on mathematical techniques, i.e., linear regression and cubic regression. The data analysis based on these mathematical techniques is presented in the following sections.

### 3.1. Linear Regression-Based Prediction Model

The parameters of linear regression calculated from the experimental data are mentioned in Table 3.

The variables needed to produce the equation for the linear regression are represented as T for the temperature and $R_{a}$ for the surface roughness, along with $\bar{T}$ and ${\bar{R}}_{a}$ for the mean values, respectively.

Here, ‘b’ represents the slope of the linear regression and is given by Equation (5):

(5) $$b=r\;\frac{S_{y}}{S_{x\;}}$$

where ‘r’ is called the Pearson’s correlation coefficient and is reflected by Equation (6):

(6) $$r=\frac{\sum \left(\left(T-\bar{T}\right)\left(R_{a}-{\bar{R}}_{a}\right)\right)}{\sqrt{\sum {\left(T-\bar{T}\right)}^{2}}\sum {\left(R_{a}-\bar{{\bar{R}}_{a}}\right)}^{2}}=0.82$$

while $S_{x}$ and $S_{y}$ are the standard deviations of the  x and y and are given by Equations (7) and (8):

(7) $$S_{x}=\sqrt{\frac{\sum {\left(T-\bar{T}\right)}^{2}}{n-1}}=79.04$$

(8) $$S_{y}=\sqrt{\frac{\sum {\left(R_{a}-{\bar{R}}_{a}\right)}^{2}}{n-1}}=0.23$$

where ‘n’ represents the number of the inputs.

Solving for ‘b’ from Equation (5) produces Equation (9):

(9) $$b=r\;\frac{S_{y}}{S_{x\;}}=0.0023$$

Correspondingly, ‘a’, the bias coefficient and the y-intercept of the linear regression, is given by Equation (10):

(10) $$a={\bar{R}}_{a}-b\bar{T}=4.3$$

where $\bar{T}$ and ${\bar{R}}_{a}$ are the mean values of the input variable $\left(T\right)$ and output variable $\left(R_{a}\right).$

Putting these values in the general Equation (1) of the linear regression in the form of the surface roughness and temperature reflects Equation (11):

(11) $$R_{a}=0.0023\cdot T+4.3$$

Henceforth, these coefficient values, i.e., a and b, are estimated, on the basis of which the response of the system is predicted for the missing data.

### 3.2. Cubic Regression-Based Prediction Model

Cubic regression can be performed by a number of software platforms and simulating tools, i.e., MATLAB, Desmos, SegRegA and many more, as manual calculation can be hectic. In this investigation, MATLAB and Desmos were used for this purpose. The values for the regression are reflected as

$$a=3.2632\cdot 10^{-7},\;b=-0.000207556,\;c=0.043397,\;d=1.84264$$

forming the cubic regression Equation (12) in the form of surface roughness and temperature as

(12) $$R_{a}=3.2632\cdot 10^{-7}\cdot T^{3}-0.000207556\cdot T^{2}+0.043397\cdot T+1.84264$$

Using the values of these coefficients, i.e., a, b, c and d, the behavior of the system can be predicted for the unknown data.

### 3.3. The Proposed Analytical Model

**Figure 5:**
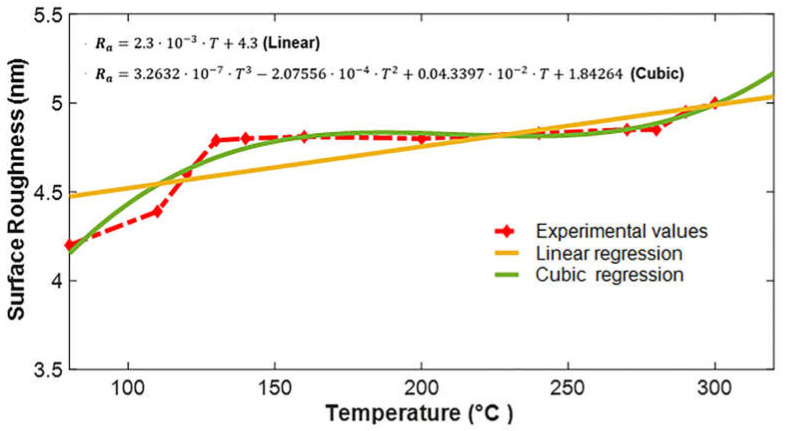
Graphical representation of the experimental data (dotted red) and analytical prediction model application of models based on linear regression (orange) and cubic regression (green).

The graphical representation of the two prediction models, i.e., linear and cubic regression, for the experimental data up to T=300 °C is shown in Figure 5. Analyzing the trends in graphs, it can be seen that the change in surface roughness due to change in the temperature up to 300 °C can be predicted by both forms of the regressions, having a similarity index of 67%−93%.

**Figure 6:**
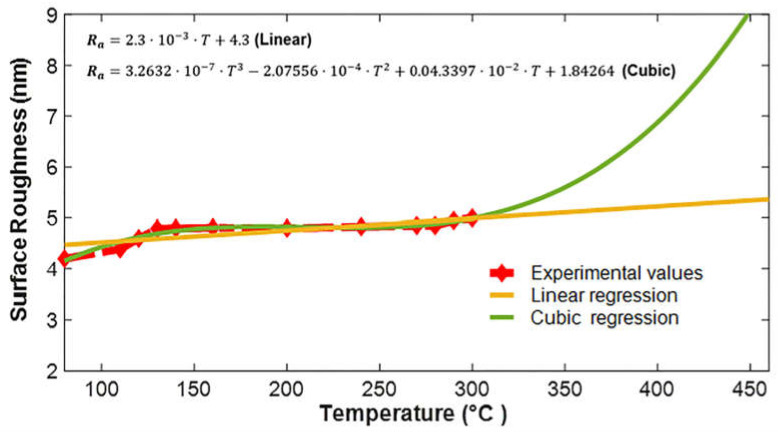
The prediction of surface roughness against rise in temperature in PECVD process by linear (orange) and cubic regression (green) for a higher temperature value, i.e., 450 °C.

**Figure 7:**
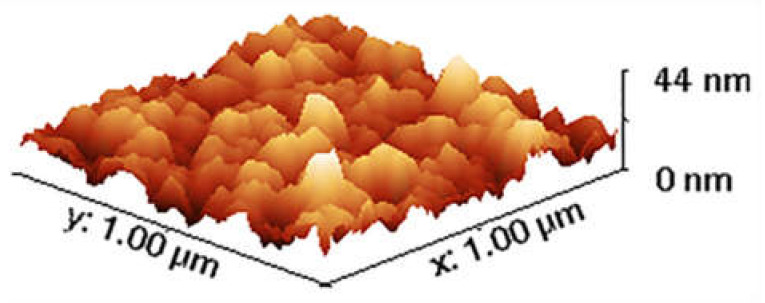
A typical AFM image of the surface roughness of a SiO_2_ layer deposited by PECVD process.

Figure 6 depicts the surface roughness prediction up to a value of T=450 °C  using the proposed analytical models based on linear and cubic regressions. Analyzing the trends in the graph, the linear regression model shows a partial agreement with the experimental data; however, it deviates completely from the given values above and below the data range. Moreover, when comparing trends of the cubic regression model, it shows a good agreement with the experimental values in the range of 80 to 300 °C. After 300 °C, it shows an upward trend in the curve, predicting an increase in the surface roughness. Similarly, a typical AFM image of the surface roughness is presented in Figure 7.

### 3.4. Error Bars and Correlation

**Figure 8:**
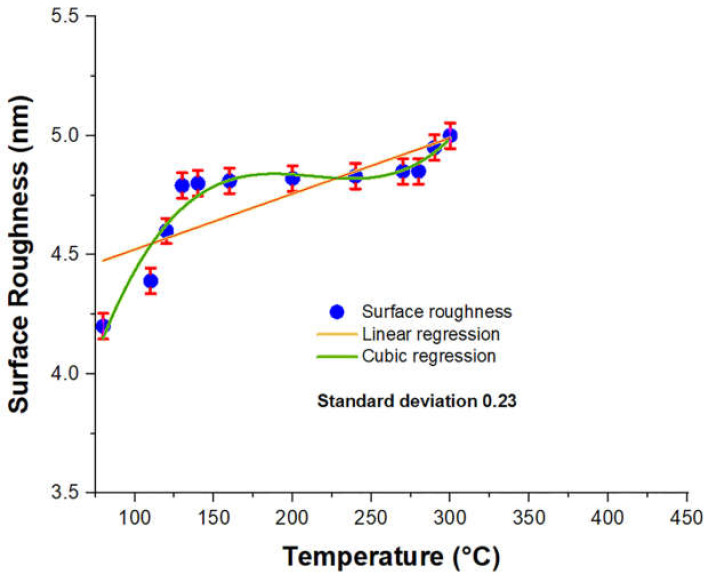
The error bars of the investigated data with the predicted data by the linear (orange) and cubic (green) regressions.

The error bars of the investigated data are considered with those of the predicted data by the machine learning algorithms, i.e., linear and cubic regressions. Moreover, the error bars are produced by calculating the standard deviation from the investigated data and reflecting them into bars with the predicted data as produced in Figure 8.

The correlations of the linear and cubic regressions between the two quantities, i.e., change in the temperature and surface roughness, are summarized in Table 4, in order to account for the similarity between the investigated data and predicted data. Moreover, they reflect the prediction of the results for the missing data up to 450 °C.

## 4. Conclusions

In this research work, a machine learning-based mathematical model was developed for the prediction of the surface roughness against change in the temperature in the PECVD thin-film deposition process. This prediction model will help scientists and researchers to readily find out the surface roughness values at a given temperature of the PECVD process without consuming time and costly resources. The prediction is based on experimental data for SiO_2_ deposition with a substrate temperature varied in the range of 80–300 °C. The experimental results for surface roughness against the varied temperatures were measured using AFM surface profiling. Based on the experimental data, two different prediction models were generated using linear regression and cubic regression. The analytical predictions showed a good agreement with the given data, and they were found useful in predicting the missing data values between the given data and above and below them. However, the behavior of the prediction models was found different from the data values above and below the experimental data ranges, which indicates an abrupt behavior of surface roughness for extreme temperature values. That is, the surface roughness might abruptly increase or fall at the temperature ranges outside the operating temperature range (80 to 450 °C) of PECVD equipment. The prediction models are compared and validated by applying correlation and calculating data error bars. Thus, it was found that the surface roughness and the change in the temperature in this investigation were not linearly attached, and their trends did deviate from each other with a moderate similarity index of 67%. Correspondingly, using the cubic regression, the spectrums did match and produced a well-performing response with the investigated data having a similarity index of about 93%.

## Figures and Tables

**Table 1:** Parameters of the PECVD process for the $S_{i}O_{2}$ deposition.

Parameters	Values
$S_{i}H_{4}N_{2}$ (sccm)	430
$NH_{3}$ (sccm)	710
$N_{2}O$ (sccm)	0
HF power (watt)	20
LF power (watt)	20
Pressure (torr)	1

**Table 2:** The change in surface roughness against the variation in the temperature using the PECVD process for the $S_{i}O_{2}$ layer deposition.

Temp vs. Surface Roughness of SiO_2_ Layers Using PECVD Process		
Sr. No.	Temperature (°C)	Surface Roughness (nm)
**1**	80.00	4.20
**2**	110.00	4.39
**3**	120.00	4.60
**4**	130.00	4.79
**5**	140.00	4.80
**6**	160.00	4.81
**7**	200.00	4.80
**8**	240.00	4.83
**9**	270.00	4.85
**10**	280.00	4.85
**11**	290.00	4.95
**12**	300.00	5.00

**Table 3:** Calculations needed to produce the predictability equation for the linear regression.

Temperature *(T)*	Surface Roughness $\left(R_{a}\right)$	$\left(T-\bar{T}\right)$	$\left(R_{a}-{\bar{R}}_{a}\right)$	$\left(T-\bar{T}\right)$ $\left(R_{a}-{\bar{R}}_{a}\right)$	${\left(T-\bar{T}\right)}^{2}$	${\left(R_{a}-{\bar{R}}_{a}\right)}^{2}$
**80**	4.20	−113.33	−0.539	61.087	12,844.369	0.291
**110**	4.39	−83.333	−0.349	29.083	6944.389	0.122
**120**	4.60	−73.333	−0.139	10.193	5377.729	0.019
**130**	4.79	−63.333	0.051	−3.238	4011.069	0.003
**140**	4.80	−53.333	0.061	−3.253	2844.409	0.004
**160**	4.81	−33.333	0.071	2.367	1111.089	0.005
**200**	4.80	6.667	0.061	0.407	44.449	0.004
**240**	4.83	46.667	0.091	4.247	2177.809	0.008
**270**	4.85	76.667	0.111	8.510	5877.829	0.012
**280**	4.85	86.667	0.111	9.620	7511.169	0.012
**290**	4.95	96.667	0.211	20.397	9344.509	0.045
**300**	5.00	106.66	0.261	27.840	11,377.849	0.068
$\bar{T}$ **=193.333**	${\bar{R}}_{a}$= 4.739	-	-	∑=167.26	∑=69021.66	∑=0.593

**Table 4:** The calculated correlations of the linear and cubic regressions.

Linear Correlation (r^2^)	Cubic Correlation (r^2^)
0.64008	0.9321
Moderate correlation	Strong correlation

## Data Availability

The authors confirm that the data supporting the findings of this study are available within the article.
